# Influence of Composition and Network Formation Sequence on the Responsive Behavior of Double-Network Hydrogels

**DOI:** 10.3390/gels12030260

**Published:** 2026-03-21

**Authors:** Lenka Hanyková, Julie Šťastná, Ivan Krakovský

**Affiliations:** Department of Macromolecular Physics, Faculty of Mathematics and Physics, Charles University, V Holešovičkách 2, 180 00 Prague, Czech Republic; chamky@seznam.cz (J.Š.); ivank@kmf.troja.mff.cuni.cz (I.K.)

**Keywords:** thermoresponsive hydrogel, double network, poly(*N,N’*-diethylacrylamide), NMR spectroscopy

## Abstract

This study investigates how the composition and synthesis sequence affect the structure and responsive behavior of single-network (SN) and double-network (DN) hydrogels composed of poly(*N,N’*-diethylacrylamide) (PDEAAm) and polyacrylamide (PAAm). DN hydrogels were prepared in two configurations, PDEAAm/PAAm and PAAm/PDEAAm, and compared with SN copolymer hydrogels of varying DEAAm/AAm ratios. ^1^H NMR spectroscopy revealed that DN hydrogels exhibit significant heterogeneity due to polymer-rich domains, impacting the accuracy of compositional determination and leading to broad NMR signals. Temperature-dependent NMR and gravimetric swelling analyses were used to quantify thermoresponsive behavior, showing that SN copolymer hydrogels exhibit composition-dependent phase transition parameters, while DN hydrogels show relatively constant transition parameters due to heterogeneous structures. NMR relaxation studies of water molecules identified “free” and “bound” molecules whose dynamics differ markedly above the transition temperature, especially in DN systems. Finally, the swelling behavior in water–acetone mixtures was examined, revealing distinct responses depending on hydrogel composition and thermal state. PAAm-rich hydrogels showed abrupt deswelling near 40 vol% acetone, while PDEAAm-based hydrogels responded more gradually. The findings demonstrate that both composition and network formation order critically influence the structural, thermal, and solvent-responsive properties of hydrogels, offering insights for the design of stimuli-responsive materials.

## 1. Introduction

Hydrogels, three-dimensional polymer networks that can absorb and retain large volumes of water, have attracted considerable interest in materials science due to their unique characteristics and wide-ranging applications. Their porous, hydrophilic structures closely mimic the properties of natural living tissues, making them ideal for uses in drug delivery, tissue engineering, and biosensing [1,2,3]. Among hydrogels, responsive hydrogels—which undergo reversible structural or property changes in reaction to external stimuli—are especially promising. These stimuli may include pH shifts, temperature changes, light, or specific molecules. This adaptability allows responsive hydrogels to dynamically adjust to varying conditions, making them highly valuable in applications requiring precise material control [4,5,6,7].

Conventional hydrogels often lack sufficient mechanical strength, limiting their applications. Various strategies have been employed to improve their mechanical strength, resulting in new categories such as topological hydrogels [8,9], hybrid hydrogels [10,11], nanocomposite hydrogels [12,13] and double network (DN) hydrogels [14,15,16]. Among these, DN hydrogels are especially notable for their enhanced mechanical strength and high fracture toughness. DN hydrogels are a unique type of interpenetrating network hydrogel, comprising two networks with contrasting properties, such as differences in crosslinking density, rigidity, and molecular weight. The toughening mechanism of DN hydrogels relies on this contrast: during deformation, the stiff and brittle first network bears the majority of the stress, while the softer second network dissipates energy around crack tips, preventing rupture and enhancing durability [17,18].

Poly(*N*-isopropylacrylamide) (PNIPAAm) is often used as a temperature-sensitive component in DN hydrogels. Early studies explored nanocomposite DN PNIPAAm hydrogels with polysiloxane nanoparticles, enhancing deswelling, stiffness, and strength [19]. Ultra-tough hydrogels were synthesized using poly(2-acrylamido-2-methylpropane sulfonic acid) as the first network and PNIPAm copolymerized with zwitterionic [2-(methacryloyloxy)ethyl]dimethyl(3-sulfopropyl)ammonium hydroxide as the second network [20]. DN PNIPAAm/polyvinyl alcohol (PVA) hydrogels, designed to enhance fracture toughness, were prepared by modifying PVA with methacrylic acid and copolymerizing with PNIPAAm via photoinitiation in a 3D-printed mold [21].

Although DN hydrogels are widely recognized for their outstanding mechanical properties, understanding their microscopic structural behavior is essential for the rational design and further development of these materials as mechanically robust hydrogels. The present study focuses primarily on their microscopic structural behavior and temperature-induced polymer mobility, investigated by NMR spectroscopy and swelling measurements. In previous research, we focused on studying DN hydrogels prepared from temperature-sensitive PNIPAAm or poly(*N,N’*-diethylacrylamide) (PDEAAm) [22]. The findings revealed that the preparation parameters of the first network, such as the type and concentration of monomers or crosslinking density, are critical for the subsequent step in the synthesis of DN hydrogels. During this step, the first network is swollen with the monomer of the second network, and this swelling significantly influences the polymerization process, ultimately affecting the final composition and properties of the DN hydrogel. Building on these results, we aim to further explore how preparation parameters influence the properties of DN hydrogels. In this study, we have prepared DN hydrogels by combining PDEAAm and polyacrylamide (PAAm) in different sequences, namely PDEAAm/PAAm and PAAm/PDEAAm, and at varying polymer concentrations. For comparison, single-network (SN) hydrogels with different copolymer compositions of P(DEAAm-co-AAm) will also be analyzed.

## 2. Results and Discussion

### 2.1. Hydrogels Composition

Three types of hydrogel systems were prepared: single-network (SN) hydrogels, PDEAAm and PAAm; double-network (DN) hydrogels combining PDEAAm and PAAm in different sequences, PDEAAm/PAAm and PAAm/PDEAAm; and copolymer hydrogels P(DEAAm-co-AAm), with varied monomer composition. An overview of the measured samples is given in Table 1.

**Figure 1:**
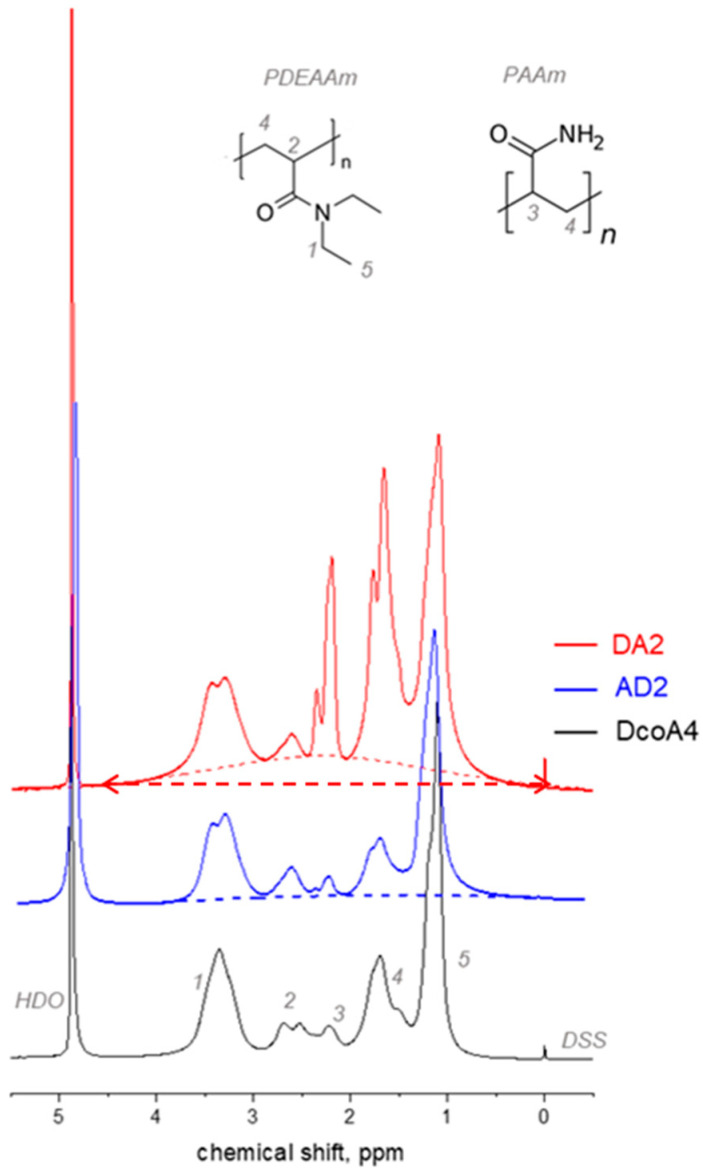
^1^H NMR spectra of hydrogels recorded at 290 K. NMR signals are assigned to PDEAAm and PAAm polymer groups. The wide component, as discussed in the text, is marked with a dashed line and the corresponding half-width with an arrow.

Figure 1 displays ^1^H spectra acquired from DA2, AD2 and DcoA4 hydrogels, with NMR signals assigned to polymer groups of PDEAAm and PAAm. Signals at 4.94 ppm and 0 ppm correspond to residual water molecules HDO and standard DSS, respectively. Upon initial observation, it is evident that the DA2 hydrogel spectrum exhibits two distinct components: narrow signals (width ≈ 0.1–0.5 ppm) and a broader component (width ≈ 4.5 ppm), which is marked with the dashed line. This broad signal was observed for all PDEAAm/PAAM DN hydrogels and indicates the formation of heterogeneous structures during the synthesis of these hydrogels. Within these structures, certain polymer-rich regions (domains) exhibit restricted mobility of the polymer chains, which subsequently give rise to the broad component observed in the NMR spectra. Similar heterogeneous regions were also observed in DN PNIPAAm/PAAm hydrogels [23]. On the other hand, in the case of PAAm/PDEAAm hydrogels, the broad signal makes a much lesser contribution, and their spectra bear greater resemblance to the spectra of copolymer SN hydrogels. The differing behavior of PDEAAm/PAAm and PAAm/PDEAAm hydrogels may be attributed to the greater swelling capacity of the PAAm SN hydrogel compared to PDEAAm, as well as the liquid state and high solubility of the DEAAm monomer. As a result, networks formed in the PAAm/PDEAAm sequence exhibit less heterogeneity than those in the PDEAAm/PAAm sequence.

**Figure 2:**
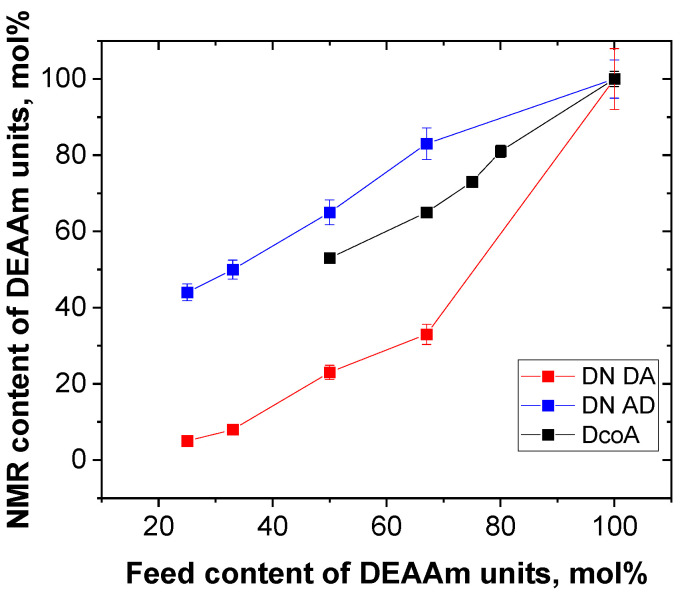
Dependence of molar content of DEAAm units from NMR spectra on feed molar content of DEAAm units.

The integral intensities of the NMR signals were used to determine the values of the molar ratio of polymerized DEAAm and AAm in the hydrogels. For PDEAAm/PAAm DN hydrogels, the baseline was corrected to eliminate the broad signal in the spectra. The values of the molar ratio of polymerized DEAAm and AAm in hydrogels were determined using the relation

(1) $${\left(\frac{N_{DEAAm}}{N_{AAm}}\right)}_{NMR}=\frac{3I_{1}}{4\left(I_{2}+I_{3}+I_{4}+I_{5}\right)-9I_{1}}$$

where *I_i_* is the integrated intensity of the signal *i* marked in the spectra in Figure 1. Table 1 contains the values of the molar ratios (*N*_DEAAm_/*N*_AAm_)_NMR_ calculated using this method. Simultaneously, Figure 2 illustrates the relationship between the feed molar content of DEAAm units and the molar content of DEAAm units as determined from NMR spectra.

Table 1 and Figure 2 provide evident support for the fact that the composition of the copolymer SN hydrogels, as deduced from the NMR spectra, closely matches the composition of the DEAAm and AAm monomers in the reaction mixture used during synthesis, within the acceptable margin of error. In contrast, the molar ratios (*N*_DEAAm_/*N*_AAm_)_NMR_ and (*N*_DEAAm_/*N*_AAm_)_feed_ of both types of DN hydrogels differ from each other, so that the molar NMR content is higher than the feed content for the component that was incorporated into the DN as the second (Figure 2). This finding is unexpected because one might assume that the first highly cross-linked network component would cause less swelling of the second monomer, resulting in a smaller NMR content compared to the feed content. However, the presence of a broad signal in the NMR spectrum (Figure 1) provides an explanation for this phenomenon. The polymer units of the first component, present in the polymer-rich domains and contributing to the broad signal, do not participate in the narrow NMR signals, leading to their exclusion from the molar ratio (*N*_DEAAm_/*N*_AAm_)_NMR_ (Table 1). This effect is more pronounced in the case of PDEAAm/PAAM DN hydrogels, leading to a more significant difference between their NMR and feed molar ratios.

In the following text and figures, we present the DEAAm/AAm ratio in DN hydrogels as determined by NMR.

### 2.2. Temperature-Sensitive Behavior

#### 2.2.1. ^1^H NMR Spectroscopy

**Figure 3:**
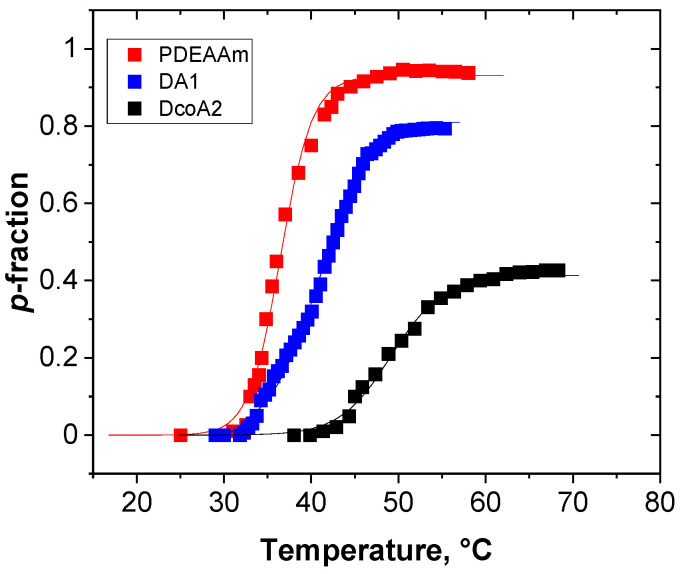
Temperature dependence of *p*-fraction of DEAAm diethyl CH_2_ group detected for various hydrogels.

For quantitative description of changes in NMR spectra, the *p*-fraction (degree of collapsing) was used, which is introduced in Section 4.3 (Equation (4)). Figure 3 illustrates how the *p*-fraction of the DEAAm CH_2_ signal varies with temperature for three specific samples. Evidently, in contrast to the PDEAAm hydrogel featuring an SN network, hydrogels incorporating AAm units either as part of a copolymer chain or within a DN exhibit temperature-related alterations in the NMR spectra at elevated temperatures. Concurrently, the NMR signals corresponding to DEAAm units remain detectable, unlike the scenario observed in the SN PDEAAm hydrogel, where the *p*-fraction approaches 1, indicative of complete signal disappearance.

The *p*-fraction’s sensitivity to temperature was modeled through the utilization of Equation (2), as previously developed [24].

(2) $$p\left(T\right)=\frac{p_{max}}{1+exp\left(\frac{{∆H}_{NMR}}{RT}-\frac{{∆S}_{NMR}}{R}\right)}$$

**Figure 4:**
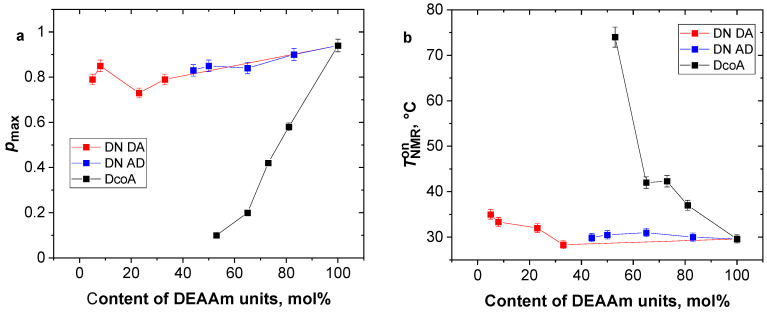
Correlation between parameters *p*_max_ (**a**) and $T_{NMR}^{on}$ (**b**) as determined by fitting Equation (2), with respect to the content of PDEAAm units. Data represent mean values obtained from three independent samples; error bars indicate standard deviations.

Here, *p*_max_ represents the uppermost limit of the *p*-fraction, while Δ*H*_NMR_ and Δ*S*_NMR_ correspond to alterations in enthalpy and entropy linked to the phase transition. In this equation, *R* signifies the universal gas constant and *T* stands for the absolute temperature. The NMR-derived transition temperatures, denoted as $T_{NMR}^{on}$, were determined by identifying the initiation point of the sigmoidal *p*(T) curve. This was achieved by locating the point where the tangent at the inflection point of the *p*(T) curve intersects the *x*-axis. All measurements were performed on three independently prepared hydrogel samples. The parameters *p*_max_ and $T_{NMR}^{on}$ were obtained by fitting Equation (2) to the experimental *p*(T) dependence for each replicate separately, and the values reported in Figure 4 represent mean values with standard deviations.

Consequently, the resulting fitted parameters enable a comparative analysis of the hydrogels under investigation, shedding light on their microscopic responses as the temperature rises. Within this context, the $T_{NMR}^{on}$ parameter conveys insights into the temperature initiating polymer chain aggregation, while the *p*_max_ parameter characterizes the extent of unit involvement within the aggregates (globules). Figure 4 displays the temperature dependence of these two parameters for all examined hydrogels.

Copolymer SN hydrogels exhibit a notable correlation between the parameter *p*_max_ and their composition. As the content of temperature-sensitive PDEAAm units decreases, there is a corresponding reduction in their involvement in collapsed globular structures. For instance, in the case of the DcoA2 sample containing 73 mol.% PDEAAm, the value of *p*_max_ is 0.42, indicating that only 42% of the PDEAAm units participate in the collapsed structures. Likewise, the $T_{NMR}^{on}$ value demonstrates a pronounced reliance on the composition of PDEAAm units. In the case of a pure PDEAAm SN hydrogel, this critical temperature is around 30 °C. However, when the hydrogel contains 53 mol.% PDEAAm, the $T_{NMR}^{on}$ value increases to 74 °C. Notably, the corresponding *p*_max_ value is merely 0.1, indicating a minimal alteration in the polymer chain behavior. This pattern is characteristic of a random copolymer chain, where the presence of hydrophilic PAAm units hinders the collapsing of neighboring PDEAAm units, compelling them to behave in a “hydrophilic” manner [25].

In contrast to the behavior exhibited by copolymer hydrogels, DN hydrogels demonstrate minimal alterations in $T_{NMR}^{on}$ and *p*_max_ parameters. This can be attributed to the formation of heterogeneous regions during the preparation of DN hydrogels, as elucidated in Figure 1. Polymer units residing within these polymer-rich regions exhibit limited mobility and thus do not manifest in high-resolution NMR spectra. Consequently, the *p*-factor exclusively impacts mobile polymer units, effectively collapsing nearly all of them, resulting in pmax values ranging from 1 to 0.8 across all DN hydrogels. Simultaneously, the aggregation of mobile polymer units occurs within the temperature range of $T_{NMR}^{on}$, which spans from 30 to 35 °C. This observation suggests that temperature-sensitive PDEAAm units remain virtually unaffected by the presence of PAAm units, whether they constitute the second or first network in PDEAAm/PAAm and PAAm/PDEAAm hydrogels, respectively.

#### 2.2.2. Swelling Experiments

**Figure 5:**
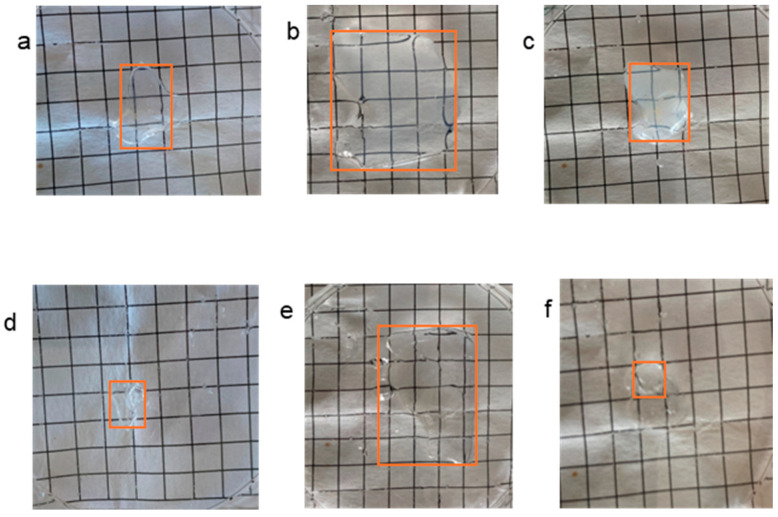
Photographs of hydrogels DcoA1 (**a**–**c**) and DA1 (**d**–**f**) in a dry (**a**,**d**), swollen (**b**,**e**) and collapsed state (**c**,**f**). The edges of the sample are color-highlighted to enhance visibility.

For illustration purposes, Figure 5 shows photographs of hydrogels DcoA1 and DA1 in a dry, swollen and collapsed state.

**Figure 6:**
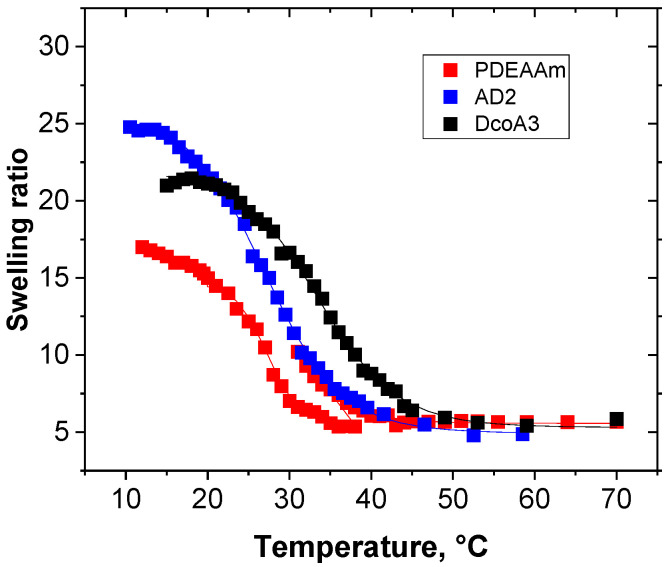
Temperature dependence of swelling ratio for various hydrogels.

Figure 6 illustrates how the swelling ratio of selected hydrogels changes in response to temperature variations. As the temperature rises, the swelling ratio diminishes because water is released from the hydrogel structures. To model this temperature-dependent swelling ratio I(T), we employed Equation (3), originally derived in the references [22]:

(3) $$s\left(T\right)=\frac{s_{u}+s_{l}e^{-\frac{∆H_{grav}-T∆S_{grav}}{RT}}}{1+e^{-\frac{\Delta H_{grav}-T\Delta S_{grav}}{RT}}}$$

**Figure 7:**
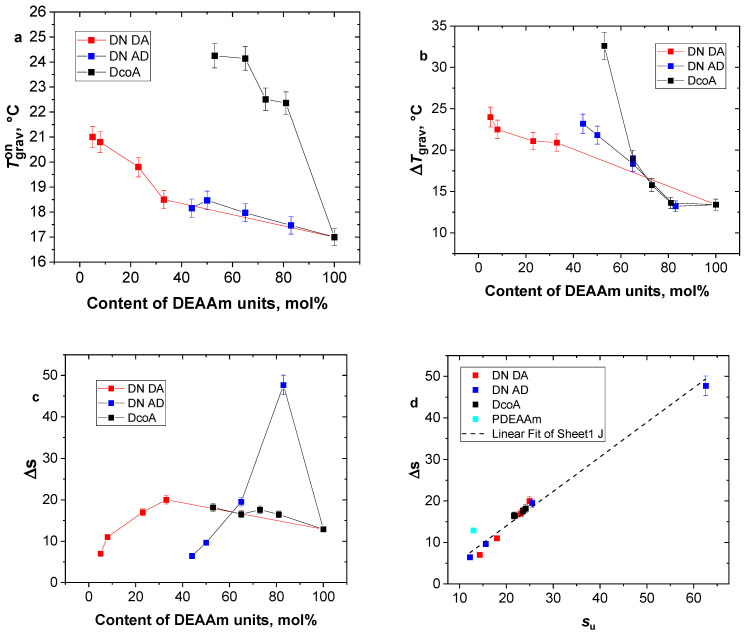
The dependence of parameters $T_{grav}^{on}$ (**a**), Δ*T*_grav_ (**b**) and Δ*s* (**c**) on the contents of DEAAm units. The correlation between Δ*s* and *s*_u_ (**d**) was fitted using linear regression. Data represent mean values obtained from three independent samples; error bars indicate standard deviations.

In this equation, Δ*H*_grav_ and Δ*S*_grav_ are the standard change in enthalpy and entropy, respectively, which are determined through gravimetric measurements. The parameters *s*_u_ and *s*_l_ represent the upper and lower limits, respectively, of the *s*(*T*) curve. By utilizing these parameters, we can ascertain the extent of deswelling as the difference Δ*s* = *s*_u_ − *s*_l_. The onset temperature $T_{grav}^{on}$ was determined similarly to the case of NMR experiments. The offset temperature $T_{grav}^{off}$ is determined at the crosspoint of the tangent with the *s*_l_ line. The width of the phase transition can be determined as Δ*T*_grav_ = $T_{grav}^{on}\;-\;T_{grav}^{off}$. All measurements were performed on three independently prepared hydrogel samples. The parameters were obtained by fitting Equation (3) to the experimental dependence for each replicate separately, and the values reported in Figure 7 represent mean values with standard deviations.

Figure 7 shows the influence of the hydrogel composition (content of DEAAm units) on $T_{grav}^{on}$ Δ*T*_grav_ and Δ*s* parameters. The $T_{grav}^{on}$ parameter’s dependency (Figure 7a) exhibits similar characteristics to that of $T_{NMR}^{on}$, which is determined from the temperature-dependent p-factor. Copolymer hydrogels display a significant sensitivity to DEAAm content. In the case of DN hydrogels, PDEAAm/PAAm and PAAm/PDEAAm, the temperature $T_{grav}^{on}$ varies only within a range of approximately 5 °C. Figure 7b demonstrates the relationship between parameter Δ*T*_grav_ and hydrogel composition. It becomes evident that an increased proportion of hydrophilic AAm units not only shifts the Δ*T*_grav_ temperature to higher values but also broadens the transition between the swollen and collapsed states.

As for the Δ*s* parameter, which represents the reduction in swelling ratio during temperature increase, it does not seem to have a correlation with hydrogel composition (Figure 7c). Figure 7d, on the other hand, illustrates the relationship between Δ*s* and *s*_u_ values, where *s*_u_ represents the swelling ratio at low temperatures. This relationship appears to be distinctly linear for all the measured hydrogels, except for the pure PDEAAm hydrogel. This implies that the method used to prepare the hydrogels affects their equilibrium swelling and subsequently determines how much water is released from the hydrogel structures as the temperature rises.

Table 2 compares the $T_{NMR}^{on}$ and $T_{grav}^{on}$ values determined from NMR and swelling experiments, respectively. It is evident that the $T_{NMR}^{on}$ values are significantly higher than the $T_{grav}^{on}$ values for all samples. This difference arises from the distinct physical processes detected by the two methods. Swelling experiments identify the temperature at which the hydrogel begins to release water, whereas NMR detects the formation of compact polymer structures. The onset of polymer aggregation occurs at higher temperatures than the initial release of water from the hydrogel network.

### 2.3. NMR Relaxation of Water Molecules

While the dynamic behavior of solvent molecules, particularly water, is not commonly explored in relation to the stimuli-responsive properties of hydrogels, it has been revealed that techniques such as NMR relaxation experiments can establish a link between the microscopic dynamics of water molecules and the polymer chains’ capacity to alter their conformation. This, in turn, affects the hydrogel’s ability to transition from a swollen to a fully or partially collapsed state [26].

**Figure 8:**
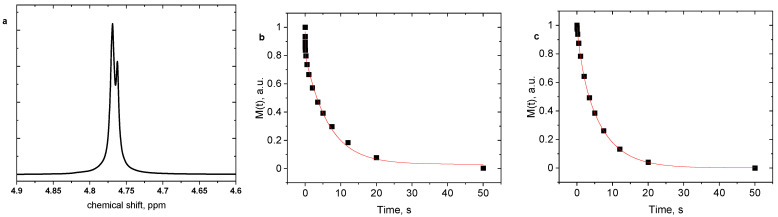
Water (HDO) signals in ^1^H NMR spectra of the hydrogel AD1 measured at 25 °C (**a**). Spin-spin relaxation curves detected at 55 °C for hydrogels PDEAAm (**b**) and AD2 (**c**) and fitted with bi-exponential dependences.

Figure 8a illustrates the D_2_O signal in the ^1^H NMR spectrum, displaying two distinct peaks. According to [27], these peaks are attributed to free water outside the hydrogel sample and bound water molecules within the hydrogel structures. The chemical environments of “free” and “bound” water molecules differ significantly, resulting in distinct chemical shifts in the NMR signals. The processing of relaxation experiments complicates chemical exchange when water molecules undergo dynamic changes between “free” and “bound” states. The assignment of “free” and “bound” water is based on the large difference in the measured transverse relaxation times. The long relaxation component *T*_2F_ (several seconds) corresponds to highly mobile water with dynamics close to bulk water, whereas the shorter component (*T*_2B_) reflects water molecules whose mobility is restricted by interactions with the polymer network. Because water molecules can rapidly exchange between different microenvironments within the hydrogel, the measured *T*_2B_ values represent exchange-averaged relaxation times rather than strictly separated water populations.

The existence of two water states is evident in the relaxation curves, displaying a bi-exponential decay with characteristic relaxation times *T*_2F_ and *T*_2B_ for “free” and “bound” water molecules, respectively, as illustrated in Figure 8b,c for PDEAAm and AD2 hydrogels. At temperatures below the transition temperature, water molecules interact with the amide groups of polymer chains mainly through hydrogen bonding, which stabilizes the hydrated polymer conformation. With increasing temperature, partial disruption of these polymer–water hydrogen bonds occurs, while hydrophobic interactions between the alkyl groups of PDEAAm units become more significant, promoting polymer aggregation and the formation of collapsed structures. In addition, hydrogen bonding between neighboring amide groups may contribute to the stabilization of these aggregated domains [28]. These temperature-dependent changes in intermolecular interactions influence the mobility of water molecules confined within the hydrogel network and are reflected in the observed *T*_2B_ relaxation times.

Table 3 presents the spin-spin relaxation time values of water, measured on selected hydrogels at temperatures of 20 and 55 °C, i.e., below and above the critical temperature, respectively. In all examined samples, mobile “free” water was observed at both temperatures, with a relaxation time *T*_2F_ ranging from 5.7 to 9 s, irrespective of temperature. The shorter relaxation time *T*_2B,_ indicative of less mobile water molecules, varied between 1.7 s and 8 ms, depending on the temperature and type of hydrogel.

**Figure 9:**
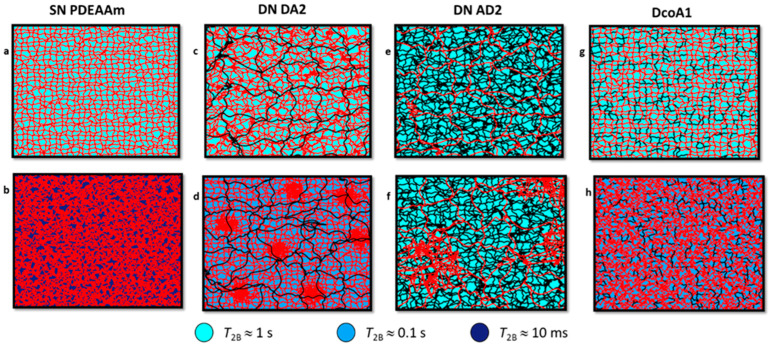
Sketch of hydrogels structure for SN PDEAAm (**a**,**b**), DN DA2 (**c**,**d**), DN AD2 (**e**,**f**) and DcoA1 (**g**,**h**) at temperatures below (**a**,**c**,**e**,**g**) and above (**b**,**d**,**f**,**h**) transition temperature. Distinct dynamic states of water molecules, characterized by varying *T*_2B_ relaxation times, are marked.

In light of the discussions in Figure 1 and Figure 2 concerning the formation of heterogeneities during the DN hydrogel preparation process and the discourse on the dynamic behavior of water, a schematic representation outlining potential structures within the investigated hydrogels was developed (Figure 9). At 20 °C, the value of *T*_2B_ relaxation time was consistently detected around 1 s for all examined hydrogels, suggesting that in equilibrium-swollen hydrogels, water exhibits a uniform relaxation behavior reflective of its interactions with the hydrogel (Figure 9a,c,e,g). In contrast, the *T*_2B_ relaxation time measured at 55 °C exhibited significant variation. For the SN PDEAAm hydrogel, the shortest *T*_2B_ time (8 ms) was recorded, and this duration is typically associated with collapsed polymer units [27], implying that, in this case, water molecules have a similar behavior to these polymer units, resulting in a substantial restriction of their mobility (Figure 9b).

At 55 °C, the *T*_2B_ relaxation times for DA2 and DcoA1 hydrogels were in the order of tenths of a second. This indicates the presence of water bound to collapsed polymer structures in these hydrogels. However, due to the coexistence of non-collapsed PAAm units, water is distributed unevenly in the measured sample, with water-rich and water-poor regions containing more and less mobile water molecules, respectively. As a result of the chemical exchange, *T*_2B_ then reflects the average relaxation times of all water molecules within the hydrogel and is therefore an order of magnitude higher than in the SN hydrogel (Figure 9d,e).

In the case of hydrogel AD2, where the hydrophilic PAAm network was prepared first, similar *T*_2B_ relaxation values were observed at both low and high temperatures. This suggests a relatively high mobility of water molecules within the collapsed structures, which is probably a consequence of the formation of a collapsed polymer structure, where the differences between water-rich and water-poor areas are not distinct. Consequently, the chemical exchange-averaged water dynamics in collapsed AD2 structures closely resemble those observed in the swollen hydrogel (Figure 9f).

### 2.4. Effect of Solvent Composition

**Figure 10:**
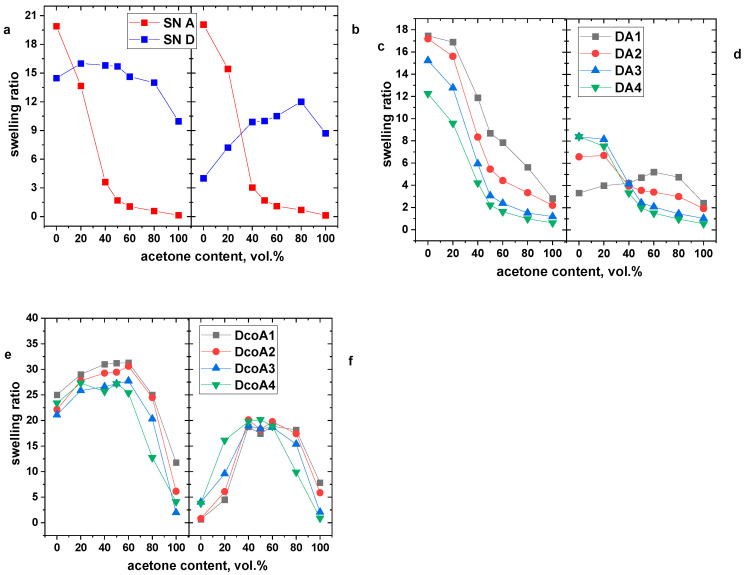
Dependence of the swelling ratio on the acetone content for SN hydrogels PAAm and PDEAAm (**a**,**b**), PDEAAm/PAAm DN hydrogels (**c**,**d**), SN copolymer hydrogels (**e**,**f**) measured at 20 °C (**a**,**c**,**e**) and at 50 °C (**b**,**d**,**f**).

We investigated how the composition of a water–acetone mixed solvent affects the swelling behavior of hydrogels at temperatures of 20 °C and 50 °C, corresponding to temperatures below and above the volume phase transition temperature (VPTT), respectively (Figure 10). For clarity, Figure 10a,b present the swelling behavior of simple SN networks at these temperatures. It is evident that PAAm and PDEAAm hydrogels exhibit different swelling responses to the solvent composition. PAAm hydrogels are known to swell in water but not in acetone. In a water–acetone mixture containing 40% acetone, a significant volume change occurs in polyacrylamide hydrogels. This step change is likely due to the interplay between osmotic pressure driving the swelling and polymer-solvent interactions. In addition to the dramatic change in swelling, this phenomenon is accompanied by shifts in other macroscopic properties such as transparency, opacity, and mechanical characteristics [29,30]. In contrast, PDEAAm exhibits a weak dependence of swelling on temperature. At 20 °C, the hydrogel swells slightly less in pure acetone compared to water, but shows a noticeably improved swelling in a mixed solvent. However, at 50 °C, the thermal collapse of PDEAAm in water becomes apparent, and the swelling ratio increases as acetone is added to the solvent mixture.

In the case of PDEAAm/PAAm DN hydrogels, the influence of the predominant PAAm component is evident. At a low temperature of 20 °C, the swelling ratio’s dependence on the acetone content mirrors that of the PAAm single-network hydrogel (Figure 10c). At higher temperatures, the swelling of PDEAAm/PAAm hydrogels is reduced due to thermal collapse. In pure acetone, the hydrogels exhibit minimal swelling, similar to their behavior at 20 °C (Figure 10d).

DcoA hydrogels also display swelling behavior in a water–acetone mixed solvent that resembles their majority component, PDEAAm. These hydrogels swell in pure water at low temperatures, and slightly more when acetone is added (Figure 10e). This effect is more pronounced than in the PDEAAm SN hydrogel, likely because DcoA hydrogels swell to a greater extent. In pure acetone, the swelling ratio remains low due to hydrophobic interactions between the PAAm monomer units and acetone. A noteworthy effect of solvent composition occurs at 50 °C (Figure 10f), where the hydrogels do not swell in pure water or acetone, but in water–acetone mixtures containing 40–60% acetone, the DcoA hydrogels reach a swelling ratio of approximately 20.

These results highlight how the responsive swelling properties of these hydrogels in solvent mixtures can be fine-tuned by adjusting their composition.

## 3. Conclusions

The study highlights the significant influence of hydrogel composition and synthesis order on their structural and responsive behaviors. Hydrogels with the opposite order of DN synthesis, i.e., PDEAAm/PAAm and PAAm/PDEAAm, were prepared and their behavior was compared with DcoA hydrogels with a copolymeric single network. In the synthesis process, the composition of DEAAm and AAm components was varied.

The analysis of NMR spectra revealed that the composition of copolymer SN hydrogels closely corresponds to the composition of DEAAm and AAm monomers in the reaction mixture used during synthesis. DN hydrogels exhibit significant discrepancies, likely due to the formation of polymer-rich domains in the initially prepared network. These domains also give rise to NMR signals with line widths on the order of thousands of hertz.

The temperature dependencies observed in NMR and swelling experiments were analyzed using previously derived thermodynamic models, and the parameters characterizing the responsive behavior were calculated. The *p*-fraction analysis reveals significant differences between copolymer and DN hydrogels, with copolymers exhibiting composition-dependent changes in the uppermost limit of the *p*-fraction *p*_max_ and the NMR-derived transition temperature $T_{NMR}^{on}$, while DN hydrogels maintain stable parameters, probably due to heterogeneous region formation. Similarly, swelling experiments demonstrate that copolymer hydrogels show a pronounced dependence on DEAAm content, whereas DN hydrogels exhibit minimal variation.

NMR relaxation experiments reveal the crucial role of water molecule dynamics in the phase behavior of hydrogels. The presence of “free” and “bound” water states, distinguished by their relaxation times, reflects interactions with the polymer network. While all hydrogels exhibit uniform water behavior at low temperatures, significant variations in *T*_2B_ relaxation times at high temperatures highlight differences in polymer collapse and water mobility. SN PDEAAm hydrogels show the most restricted water movement, whereas DN hydrogels exhibit heterogeneous water distribution due to the coexistence of collapsed and non-collapsed regions.

The swelling behavior of hydrogels in water–acetone mixtures is highly dependent on their composition and temperature. PAAm hydrogels exhibit a sharp volume change around 40 vol.% of acetone, while PDEAAm hydrogels show a more gradual swelling response, especially at higher temperatures. DN hydrogels predominantly follow the swelling behavior of their majority component, with PAAm-rich DN hydrogels resembling PAAm SN hydrogels and DcoA hydrogels behaving similarly to PDEAAm. At 50 °C, the hydrogels generally collapse in pure water and acetone but show enhanced swelling in mixed solvents. The observed differences in swelling responses to water–acetone mixtures further emphasize the impact of hydrogel composition on solvent interactions.

The results demonstrate that the responsive behavior of DEAAm/AAm hydrogels can be effectively tuned through network architecture and synthesis order without altering the overall chemical composition. Copolymer hydrogels exhibit a strong composition-dependent response, enabling fine adjustment of transition temperature and swelling behavior, which may be useful in applications requiring precise control of transport properties, such as controlled release systems or stimuli-responsive membranes. In contrast, DN hydrogels show more stable responsive parameters, likely due to their heterogeneous structure. This stability may be advantageous in applications where reproducible behavior under repeated temperature or solvent changes is required. The observed differences in swelling in water–acetone mixtures further indicate that these hydrogels can be designed to selectively respond to solvent composition, which may be relevant for sensing, separation, or solvent-responsive soft materials.

## 4. Materials and Methods

### 4.1. Chemicals

Monomers *N,N′*-diethylacrylamide (DEAAm) and acrylamide (AAm), crosslinker *N,N′*-methylenebisacrylamide (MBAAm), initiator ammonium persulfate (APS), photoinitiator 2-oxoglutaric acid (OGA) and catalyst *N,N,N′,N′*-tetramethylethylenediamine (TEMED) were purchased from Sigma-Aldrich, St. Louis, MO, USA.

### 4.2. Hydrogel Synthesis

#### 4.2.1. DN Hydrogels PDEAAm/PAAm with Various Molar Concentrations of the 2nd Component PAAm

The single-network (SN) hydrogel PDEAAm was synthesized via thermally activated redox polymerization. An aqueous solution containing the monomer DEAAm, crosslinker MBAAm, initiator APS and catalyst TEMED was prepared. DEAAm at mass concentration 127.2 g·L^−1^, MBAAm at 1.2 g·L^−1^, and APS at 1 g·L^−1^ were dissolved in deionized water and flushed with nitrogen for approximately 10 min. TEMED, at mass concentration 11.6 g·L^−1^, was then added to the solution, which was mixed thoroughly. Approximately 3 mL of the reaction mixture was injected between two glass plates (7 × 5 cm^2^) and placed in a refrigerator at 5 °C. After 24 h of polymerization, the hydrogel samples were washed with distilled water for three days, replacing the water every 24 h.

Four samples of double-network (DN) hydrogels, PDEAAm/PAAm, were prepared using this protocol: SN hydrogel PDEAAm specimens (approximately 3 × 3 × 0.1 cm^3^) were cut at 20 °C and immersed for 24 h in aqueous solutions containing the second monomer AAm at mass concentrations 35.5, 71.1, 142.2, and 213.2 g·L^−1^, along with MBAAm (0.154 g·L^−1^) and the photoinitiator OGA (1.46 g·L^−1^), at the same temperature. Prior to the swelling process, the prepared solutions were degassed with nitrogen for approximately 30 min. DN hydrogels were synthesized at room temperature (approximately 20 °C) by UV irradiation (365 nm, 3 h) of the swollen SN hydrogel specimens placed between two glass plates separated by a silicone rubber spacer with a rectangular void (4 × 4 cm^2^). After synthesis, the hydrogel samples were washed three times with a large volume of distilled water to remove residual unreacted reagents.

#### 4.2.2. DN Hydrogels PAAm/PDEAAm with Various Molar Concentrations of the 1st Component PAAm

Four samples of SN hydrogel PAAm were synthesized via redox polymerization using solutions containing AAm as the monomer at mass concentrations of 35.54, 71.08, 142.16, and 213.24 g·L^−1^, along with MBAAm as the crosslinking agent at 1.2 g·L^−1^, APS as the initiator at 1 g·L^−1^, and TEMED as the catalyst at 11.63 g·L^−1^, all dissolved in deionized water. Initially, all components except TEMED were dissolved in deionized water and degassed with nitrogen for approximately 10 min, after which TEMED was added. Roughly 3 mL of the resulting solution was injected into a mold formed by two glass plates separated by a 1 mm silicone rubber spacer, creating a rectangular cavity with dimensions of 7 × 5 cm^2^. To prevent microphase separation, polymerization was conducted in a refrigerator at 5 °C for 24 h. The resulting hydrogel samples were thoroughly washed in large volumes of deionized water over a 24 h period, with washing repeated three times to remove any residual unreacted chemicals.

The PAAm/PDEAAm DN hydrogels were subsequently prepared from the PAAm SN hydrogels. Specimens approximately 3 × 3 × 0.1 cm^3^ in size were cut from the SN hydrogels at 20 °C and swollen for 24 h in an aqueous solution (ca 50 cm^3^) containing DEAAm as the monomer at 127.18 g·L^−1^, MBAAm at 0.154 g·L^−1^, and OGA as the photoinitiator at 1.462 g·L^−1^. Before immersion, the solution was purged with nitrogen for approximately 30 min. The swollen specimens were then polymerized via UV irradiation (365 nm) for 3 h at room temperature (ca 20 °C), with the samples held between two glass plates separated by a silicone rubber spacer to create a rectangular void of 4 × 4 cm^2^. The resulting DN hydrogels were washed three times in large volumes of distilled water to remove any unreacted substances.

#### 4.2.3. SN Copolymer Hydrogels P(DEAAm-co-AAm) with Various DEAAm/AAm Molar Ratios

Four copolymer SN hydrogels were prepared by redox polymerization of solutions containing monomers DEAAm and AAm with molar ratios of 0.5:0.5, 0.67:0.33, 0.75:0.25, 0.8:0.2 mol·L^−1^; crosslinking agent MBAAm at 1.2 g·L^−1^; initiator APS at 1 g·L^−1^; and catalyst TEMED at 11.63 g·L^−1^ in deionized water. All ingredients, except for TEMED, were dissolved in deionized water and flushed with nitrogen for ca 10 min, after which TEMED was added. Approximately 3 mL of the solution was injected by a syringe into a mold assembled from two glassy plates separated by a ca 1 mm spacer from silicone rubber with a rectangular void of dimensions 7 × 5 cm^2^. To avoid microphase separation, polymerization was carried out in a refrigerator at 5 °C for 24 h. After preparation, the hydrogel samples were washed with a large amount of distilled deionized water for 1 day. Washing was repeated three times to remove all residual unreacted reagents.

The concentrations of DEAAm and AAm monomers used in preparing DN PDEAAm/PAAm and PAAm/PDEAAm hydrogels, as well as SN copolymer hydrogels P(DEAAm-co-AAm), were converted into preparation parameters—the feed molar ratios (NDEAAm/NAAm)feed and are shown in Table 1.

### 4.3. H NMR Spectroscopy

The ^1^H NMR spectra were recorded using a Bruker Avance 500 liquid-state spectrometer (Bruker, Karlsruhe, Germany) operating at 11.7 T, with 16 scans and a recycle delay of 20 s. Integrated intensities were calculated with the spectrometer software, achieving an accuracy of ±1%. For variable temperature experiments, the temperature was controlled with a precision of ±0.2 °C using a BVT 3000 temperature unit. Samples were equilibrated for 15 min prior to data acquisition. The ^1^H spin-spin relaxation times *T*_2_ of water molecules HDO were determined using the same instrument and the CPMG pulse sequence [31]: 90°x-(*t*_d_-180°y-*t*_d_)_n_-acquisition with *t*_d_ set to 0.5 ms, a relaxation delay of 100 s, and 8 scans. The *T*_2_ relaxation times were measured over a series of 35 values.

Quantitative characterization of changes during the heating process in hydrogels was achieved through *p*-fraction analysis (degree of collapsing), expressed as

(4) $$p\left(T\right)=1-\frac{I\left(T\right)*T}{I_{0}\left(T_{0}\right)*T_{0}}$$

where *I*(*T*) represents the integrated intensity of a specific polymer resonance at temperature *T* > *T*_0_, *I*_0_(*T*_0_) is the integrated intensity of the same resonance at the reference temperature *T*_0_, where no phase transition and thus no reduced polymer segment mobility occurs. The *T*/*T*_0_ correction term accounts for the decrease in integrated intensity with increasing absolute temperature as 1/*T*. The reference temperature *T*_0_ was selected as the point at which the signal intensity was highest, ensuring *p*(*T*_0_) = 0 [26].

### 4.4. Swelling Experiments

For temperature-dependent experiments, samples (1.5 × 1.5 × 0.1 cm^3^) were cut and swollen to equilibrium at room temperature (~20 °C) in bottles containing ~50 mL of distilled deionized water. The bottles were then placed in a thermostated bath set to an initial temperature of 12 °C and allowed to equilibrate for 2 h. The masses of the swollen samples were measured using a precise balance. Subsequently, the bottles containing the hydrogel samples and water were heated to the next temperature, equilibrated for another 2 h, and the measurements were repeated. This process was carried out at progressively increasing temperatures until a final temperature of 70 °C was reached.

To study solvent-responsive properties, hydrogel samples were immersed in 50 mL of a water–acetone mixed solvent for 24 h to reach equilibrium swelling, then removed, gently blotted to remove surface solvent, and weighed.

To determine the mass of the dry samples, *m*_dry_, the hydrogel samples were first air-dried at room temperature for one day and then vacuum-dried at 80 °C for another (one) day. The swelling ratio *s* was calculated by

(5) $$s=\frac{m-m_{dry}}{m_{dry}}$$

where *m* is the mass of the swollen hydrogel sample.

## Figures and Tables

**Table 1:** Hydrogel compositions determined from preparation (feed molar ratio) and NMR spectra (NMR molar ratio).

Sample		Feed Molar Ratio (*N*_DEAAm_/*N*_AAm_)_feed_	NMR Molar Ratio (*N*_DEAAm_/*N*_AAm_)_NMR_
SN PDEAAm	PDEAAm	1/0	1/0
SN PAAm	PAAm	0/1	0/1
DN PDEAAm/PAAm	DA1	0.67/0.33	0.33/0.67 ^(a)^
	DA2	0.50/0.50	0.23/0.77 ^(a)^
	DA3	0.33/0.67	0.08/0.92 ^(a)^
	DA4	0.25/0.75	0.05/0.95 ^(a)^
DN PAAm/PDEAAm	AD1	0.67/0.33	0.83/0.17 ^(b)^
	AD2	0.50/0.50	0.65/0.35 ^(b)^
	AD3	0.33/0.67	0.50/0.50 ^(b)^
	AD4	0.25/0.75	0.44/0.56 ^(b)^
SN P(DEAAm-co-AAm)	DcoA1	0.80/0.20	0.81/0.19 ^(c)^
	DcoA2	0.75/0.25	0.73/0.27 ^(c)^
	DcoA3	0.67/0.33	0.65/0.35 ^(c)^
	DcoA4	0.50/0.50	0.53/0.47 ^(c)^

^(a)^ Relative error 8%; ^(b)^ Relative error 5%; ^(c)^ Relative error 2%.

**Table 2:** The onset temperatures $T_{NMR}^{on}$ and $T_{grav}^{on}$ as determined by NMR and swelling experiments, respectively, for all samples.

Sample	$T_{NMR}^{on}$ (°C)	$T_{grav}^{on}$ (°C)
PDEAAm	29.6	17.0
DA1	28.3	18.5
DA2	32.0	19.8
DA3	33.3	20.8
DA4	35.0	21.1
AD1	30.0	17.5
AD2	31.1	17.9
AD3	30.5	18.5
AD4	29.9	18.2
DcoA1	37.0	22.4
DcoA2	42.3	22.5
DcoA3	42.1	24.1
DcoA4	73.9	24.3

**Table 3:** ^1^H spin-spin relaxation times (*T*_2F_, *T*_2B_) of water (HDO) in hydrogel samples measured at 20 and 55 °C. Values of *T*_2F_ and *T*_2B_ were obtained from bi-exponential relaxation decay.

	*T*_2F_, *T*_2B_ (s)	
Sample	20 °C	55 °C
PDEAAm	6.5, 1.5	8.0, 0.008
DA2	6.9, 1.7	7.9, 0.15
AD2	5.7, 0.9	6.7, 1.3
DcoA1	5.7, 1.6	6.8, 0.14

## Data Availability

The raw data supporting the conclusions of this article will be made available by the authors on request.

## Abbreviations

The following abbreviation is used in this manuscript:

NMR	Nuclear Magnetic Resonance
